# The risk of premature cardiac contractions (PAC/PVC) related to caffeine consumption among healthcare workers: A comprehensive review

**DOI:** 10.1002/hsr2.2222

**Published:** 2024-07-11

**Authors:** Maryam Abdolmaleki, Laya Ohadi, Faraz Changizi, Sara Seyed, Mina Farjam

**Affiliations:** ^1^ Department of Medicine, School of Medicine Shahid Beheshti University of Medical Sciences Tehran Iran; ^2^ Department of Medicine, School of Medicine Semnan University of Medical Sciences Tehran Iran

**Keywords:** arrhythmias, caffeine, cardiovascular, doctors, healthcare workers

## Abstract

**Background and Aims:**

Premature atrial contractions (PACs) and premature ventricular contractions (PVCs) are ectopic heart rhythm disorders with implications for cardiovascular health. This study explores the relationship between caffeine consumption and the risk of PACs and PVCs, with a focus on healthcare workers, such as doctors, nurses, pharmacists, and midwives, who often rely on caffeine to combat fatigue, especially during night shifts.

**Methods:**

A thorough review was conducted through PubMed, Scopus, Google Scholar, and Web of Science, utilizing a combination of MeSH terms and keywords. Studies examining the link between caffeine consumption and PACs and PVCs, particularly in healthcare workers, were included.

**Results:**

We found that caffeine shows various effects based on dosage and can impact arrhythmia risk. Individuals working long shifts, including healthcare professionals, are prone to increased caffeine intake, leading to higher cardiovascular risk. To mitigate these risks, tailored guidelines for caffeine consumption, flexible shift scheduling, and mental health support services are recommended. Promoting caffeine alternatives within healthcare institutions can be beneficial.

**Conclusion:**

Although caffeine may have potential benefits, its drawbacks, particularly concerning cardiovascular health, may surpass its advantages, especially when consumed in high doses. A multidisciplinary approach is crucial for healthcare workers’ well‐being and quality of patient care. Further research is required to refine and support these recommendations.

## 1 INTRODUCTION

### 1.1 PAC and PVC

Premature cardiac contractions, also known as atrial and ventricular ectopic beats, are ectopic heart rhythm disorders in the general population.^1^ They arise when ectopic impulses from the muscle fiber or fibers within the ventricles or atriums occur independently of the physiological pacemakers.^2^ Based on the origin of these ectopic beats, we classified them as premature atrial contractions (PACs) and premature ventricular contractions (PVCs).^3^ A correlation exists between frequent PACs and PVCs and a higher risk of cardiovascular events, morbidity, and mortality.^3^ Recent studies have shown that recurrent PACs are associated with increased atrial fibrillation, ischemic stroke, transient ischemic attack, and mortality in older patients without a history of AF.^4^

PACs may result from non‐cardiac (chronic and acute pulmonary disease, chronic renal failure, neurological disorders) or cardiac diseases (acute myocardial infarction, coronary artery disease, valvular heart diseases, and cardiomyopathy) As well as behavioral/other (like alcohol, theophylline, caffeine, smoking, and thyroid conditions).^2^

Possible causes of PVCs are categorized as cardiac, pulmonary, endocrinopathies, and behavioral/other factors. Cardiac causes include heart failure, acute myocardial infarction, hypertension with left ventricular hypertrophy, hypertrophic cardiomyopathy, congenital heart disease, and idiopathic ventricular tachycardia. Pulmonary causes encompass COPD, sleep apnea, pulmonary hypertension, and other pulmonary diseases. Endocrinopathies involve thyroid, adrenal, and gonadal abnormalities. Behavioral and other factors consist of nicotine, caffeine, alcohol, sympathomimetic agents (e.g., beta‐agonists, antihistamines), and illicit drugs (e.g., cocaine, amphetamines).^2^ The different etiologies of PAC and PVC are shown in Table 1.

**Table 1:** Etiologies of PAC and PVC.

Cardiac	Non‐cardiac	Behavioral/other
PAC		
Acute MI; coronary artery disease; valvular disease; cardiomyopathy	Acute and chronic pulmonary disease; chronic kidney disease; neurological disorders; thyroid disease	Alcohol; theophylline; caffeine; smoking
PVC		
Heart failure; acute MI; hypertension; HCM; congenital heart disease; idiopathic ventricular tachycardia	Pulmonary *(COPD, sleep apnea, pulmonary hypertension)*; endocrinopathies *(thyroid, adrenal, gonadal)*	Alcohol; smoking; caffeine; illicit drugs *(cocaine, amphetamines)*; sympathomimetic agents *(β‐agonists, antihistamines)*

Abbreviations: PAC, premature atrial contraction; PVC, premature ventricular contraction.

### 1.2 Caffeine

Caffeine is a chemical compound belonging to the methylxanthines group that acts as a stimulant for the central nervous system.^5^ In healthy individuals, the mean half‐life of caffeine in plasma is about 4–5 h, ranging from 2 to 10 h.^6^ Caffeine's mechanism involves blocking adenosine receptors, boosting dopamine release, and increasing alertness. It also elevates cAMP levels, leading to heightened stimulation. Additionally, it opens ion channels in muscles, releasing calcium ions for improved muscle function.^7^ Plants like *Camellia Sinensis*, Khat, Cola Tree, Guayusa, Coffee, Guarana, Cacao Tree, and Cassina Tree contain caffeine in their leaves, seeds, and fruits. The caffeine extracted from these plants is present in various products such as beverages (tea, coffee, sodas, and energy drinks), chocolate, analgesic medications, caffeine supplements, bronchodilators, and even shampoos.^8^, ^9^, ^10^

**Figure 1:**
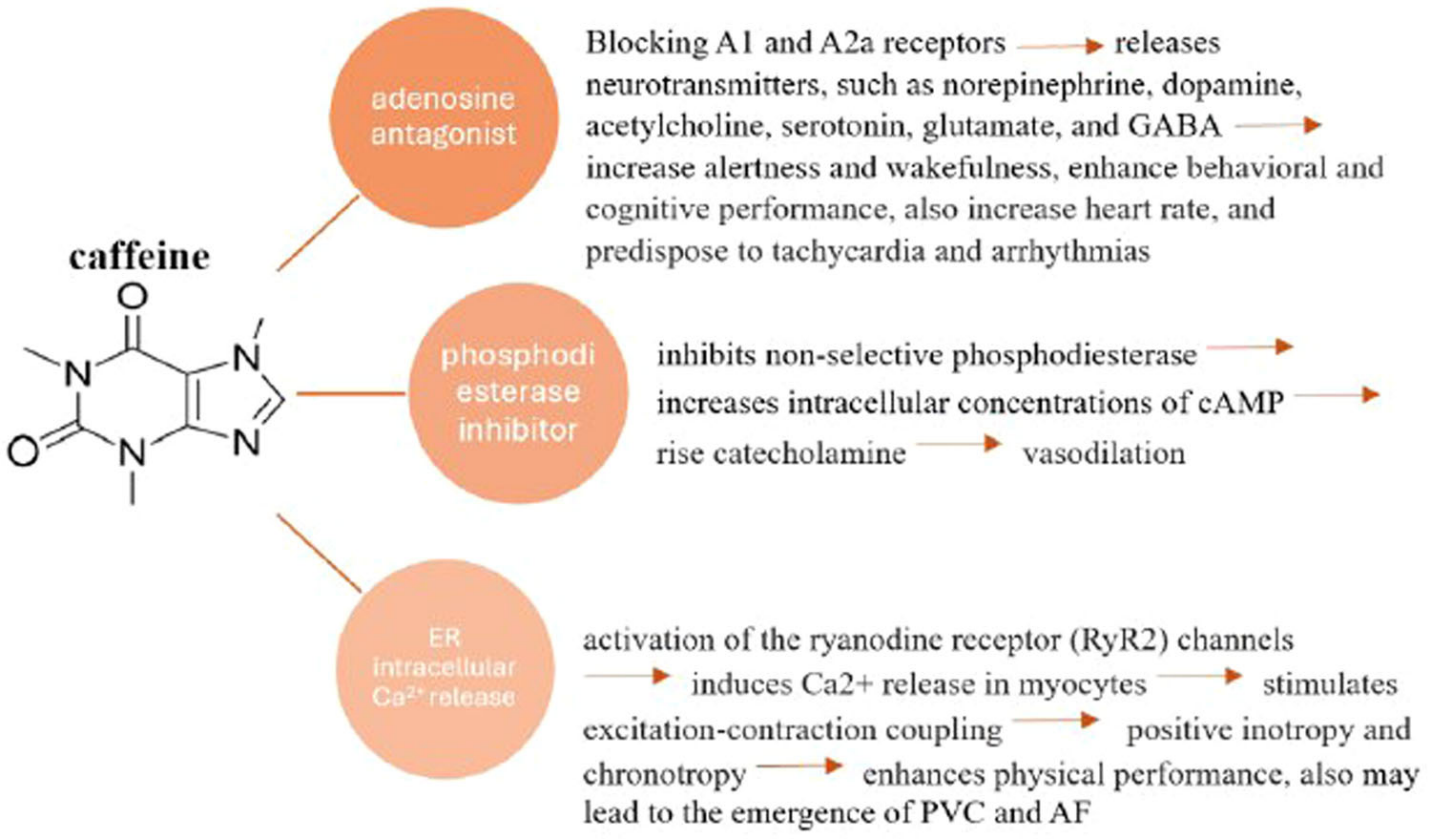
Physiological effects of caffeine on the human body. PVC, premature ventricular contraction.

Caffeine functions as an adenosine receptor antagonist with a primary focus on the A1 and A2A receptors. This interaction leads to physiological responses by partially blocking these members of the adenosine receptor family.^11^ These responses are closely intertwined with various brain functions related to sleep regulation, wakefulness, and cognitive processes. Studies have revealed that acute consumption of caffeine can notably enhance cognitive and working memory‐related brain functions in healthy adults, resulting in increased alertness, improved mood, and information processing, and enhanced attention.^12^, ^13^, ^14^ In addition, caffeine stimulates metabolic activity while reducing the sensations of tiredness and hunger, ultimately supporting the assertion that caffeine enhances overall performance.^15^ It also has positive inotropic and chronotropic effects on the cardiovascular system.^1^, ^16^ Moreover, caffeine increases norepinephrine and epinephrine plasma levels due to its sympathomimetic effects; consequently, it likely causes an increase in cardiac ectopy.^1^, ^17^ The physiological effects of caffeine on the human body are illustrated in Figure 1.

According to the guidelines, adults should limit their daily caffeine intake to 180 mg to maintain alertness and prevent health risks.^1^, ^8^ Recommended caffeine doses, based on body weight, are classified as low (3 mg/kg), moderate (5–6 mg/kg), and high (≥9 mg/kg). Up to 6 mg/kg generally has no serious adverse effects, while higher doses may lead to issues like decreased reaction time and insomnia. Athletes may require higher doses for performance enhancement. However, doses ≥9 mg/kg may not improve performance and could cause overstimulation, especially in hypersensitive individuals. Daily doses exceeding 2000 mg pose serious health risks, including hypertension and cardiac issues.^18^ Also, it can cause dependence. Abruptly stopping excessive caffeine can result in withdrawal symptoms, including headaches, palpitations, exhaustion, difficulty concentrating, and discomfort.^19^

Different caffeine‐containing drinks have different caffeine concentrations, as shown in Table 2. The numbers in the table are not exact and may vary depending on the differences in processing and brewing. Having information on caffeine concentration in different drinks reduces the risk of over‐consumption and poisoning (Staff 2022).^20^

**Table 2:** The amount of caffeine in different drinks (Staff 2022).

Drinks	Size in oz. (mL)	Caffeine (mg)
Coffee drinks		
Brewed	8 (237)	96
Espresso	1 (30)	64
Instant coffee	8 (237)	62
Tea		
Brewed black	8 (237)	47
Brewed green	8 (237)	28
Ready‐to‐drink, bottled	8 (237)	19
Soda		
Cola	8 (237)	22
Energy drinks		
Energy drink	8 (237)	71.9
Energy shot	2 (60)	215

### 1.3 Caffeine and the risk of PAC and PVC

According to the guidelines of the American Heart Association on the management of patients with arrhythmia, they are always advised to avoid caffeine consumption.^1^, ^21^ Nonetheless, the currently available data do not provide strong evidence for the association between caffeine and PAC or PVC in the general population. Most studies examining the impact of caffeine consumption on arrhythmogenesis had reported negative results.^22^, ^23^, ^24^

### 1.4 Caffeine and the risk of PACs and PVCs in healthcare workers

Caffeine has effects on vitality, alertness, fatigue, and cognitive performance.^25^ Previous research suggests that caffeine can help shift workers prevent fatigue.^26^ Therefore, caffeine consumption has increased among shift workers, particularly among EMS and healthcare workers, in an attempt to reduce fatigue and improve performance.^25^

Due to a lack of data on the risk of PACs and PVCs associated with caffeine consumption among healthcare workers, we aimed to investigate this issue and recommend some critical points to reduce the risk of arrhythmia among healthcare professionals. Concerning the novelty of this study, we found no recent similar review studies exploring the link between caffeine consumption and the occurrence of PAC and PVC in healthcare workers.

## 2 METHODS

We conducted an extensive database search across PubMed, Scopus, Google Scholar, and Web of Science during 2 months (October and November 2023) using a combination of MeSH terms and keywords like “Caffeine,” “Atrial Premature Complexes,” “Health Personnel,” “Cardiotonic Agents,” “Heart Disease Risk Factors,” and “Arrhythmias, Cardiac.” The inclusion criteria were as follows: (1) All types of studies exploring the link between caffeine and PVC/PAC; (2) studies evaluating caffeine consumption in healthcare professionals.

## 3 EPIDEMIOLOGY

### 3.1 PAC

**Figure 2:**
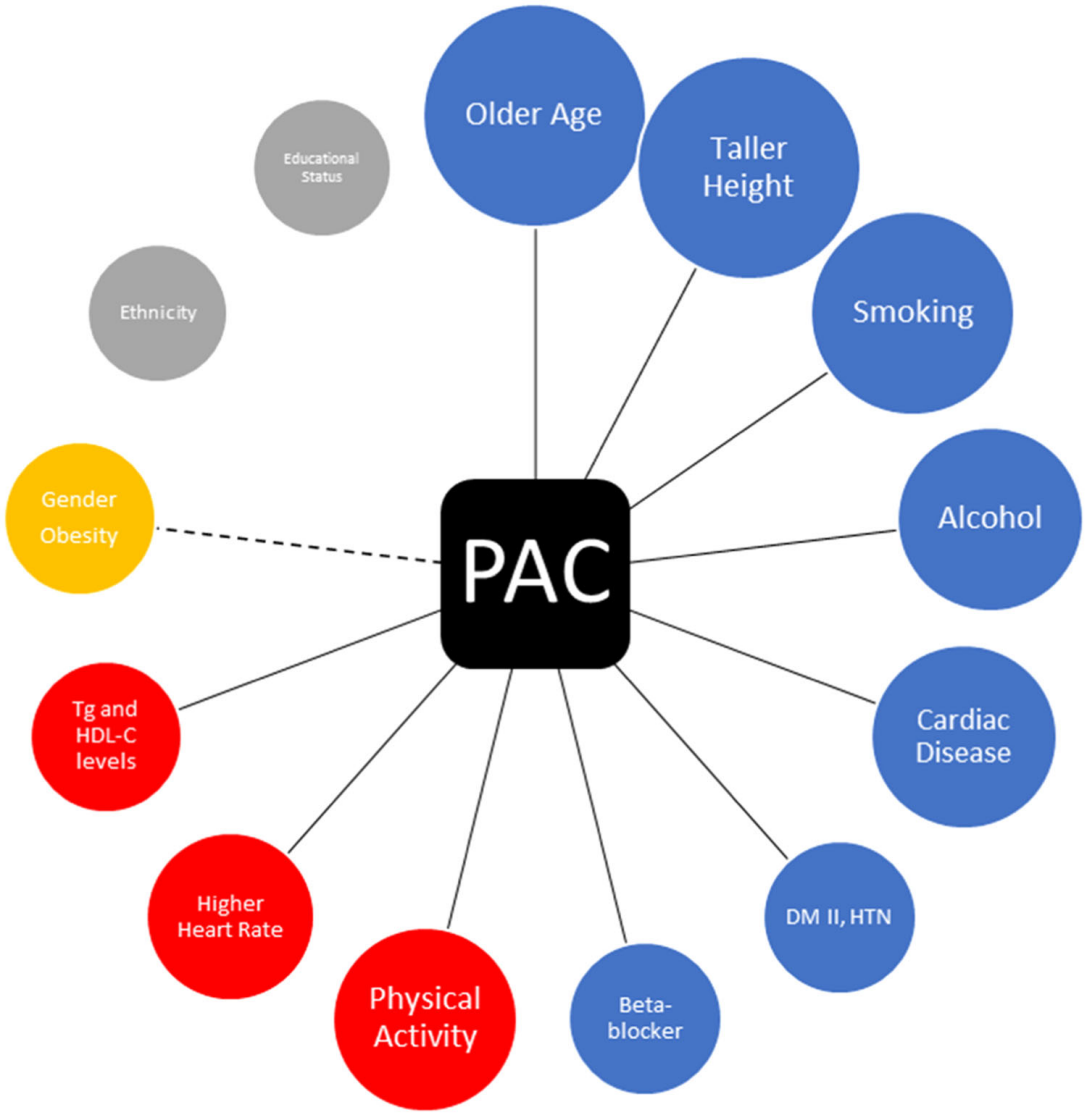
Factors associated with the occurrence of PAC. Blue circles elevate the possibility of PAC while red circles decrease the possibility. PAC, premature atrial contraction.

Studies have shown that PAC is more common than PVC in the general population. A 24‐h monitoring of men without cardiac disease aged 40–79 years in Japan showed that 99% of them had PAC within a day.^27^ Different studies agree on the relationship between age, height, smoking, alcohol consumption, higher levels of natriuretic peptide, hypertension, diabetes mellitus type II, and cardiac disease with a higher frequency of PAC.^3^, ^27^, ^28^, ^29^ Ethnicity and educational status do not significantly impact the frequency of PAC. There is conflicting evidence regarding obesity and gender. Factors associated with PVC occurrence are shown in Figure 2.

### 3.2 PVC

**Figure 3:**
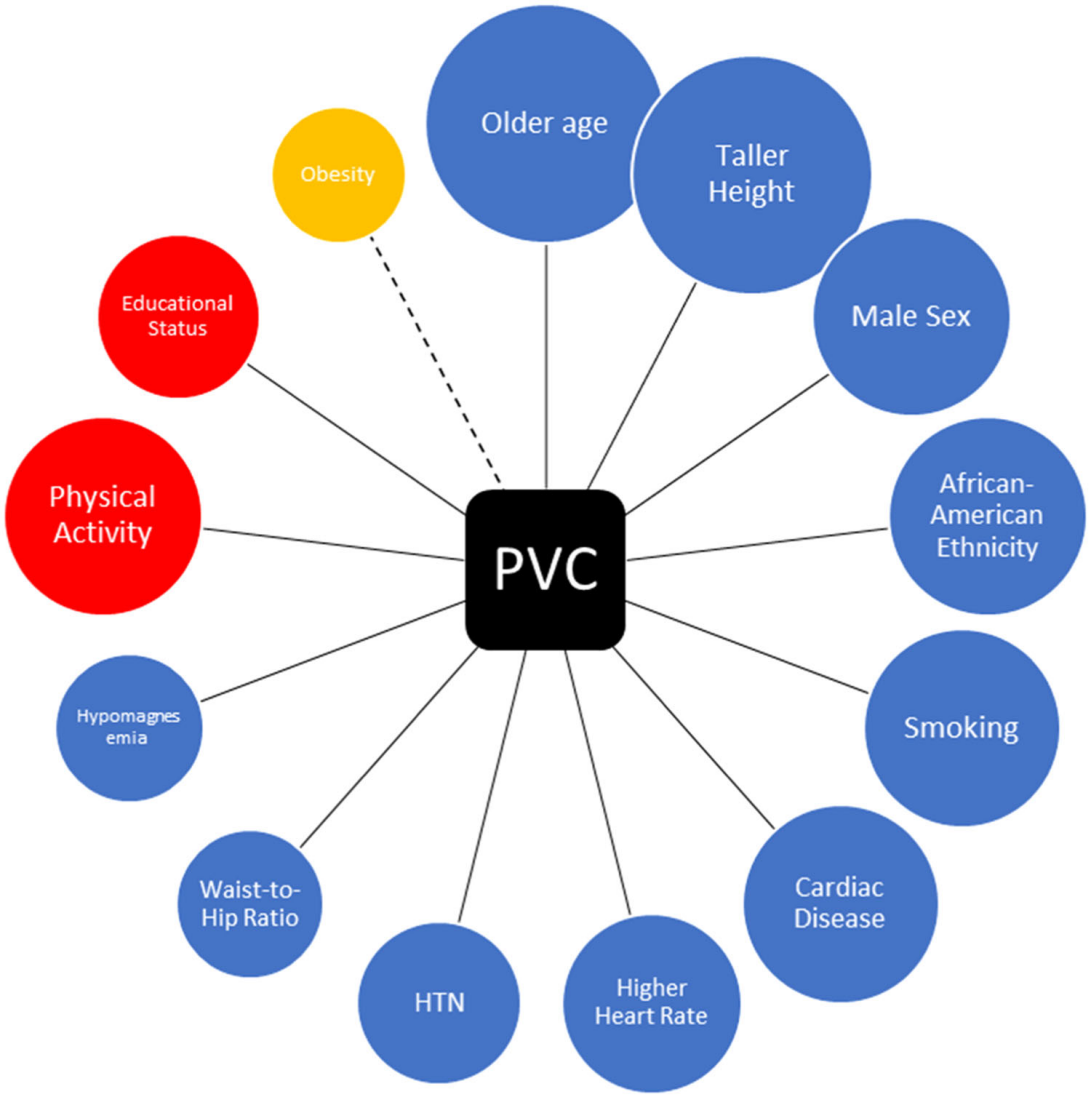
Factors associated with the occurrence of PVC. Blue circles elevate the possibility of PAC while red circles decrease the possibility. PAC, premature atrial contraction; PVC, premature ventricular contraction.

Many studies have reported different rates of PVC in different populations. It is believable that everyone experiences PVC in their lifetime at different frequencies.^30^ In a recent review by Klewer et al.,^31^ the prevalence of PVC in the general population is 3%–20%, mainly diagnosed in palpitation check‐ups or incidentally in routine health check‐ups. Some factors have been reported to be associated with higher rates of PVC, including old age, male sex, taller height, African‐American ethnicity, smoking, less physical activity, higher waist‐to‐hip ratio, hypomagnesemia, low educational status, cardiac disease, and hypertension.^32^, ^33^, ^34^, ^35^ Factors associated with the occurrence of PVC are shown in Figure 3.

The associations between various factors and PACs and PVCs are summarized in Table 3.

**Table 3:** The relationship between different factors and PAC and PVC.

Factor	Association with PACs^3^, ^27^, ^28^, ^29^	Association with PVCs^32^, ^33^, ^34^, ^35^
Age	99% increase in PACs with aging	99% increase in PVCs with aging
Diabetes mellitus type II	Correlation with PACS	Correlation with PVCs
Cardiac disease	Higher frequency of PACS	Higher frequency of PVCS
Ethnicity	No significant impact on PAC frequency	Higher PVC frequency in African‐American ethnicity
Educational status	No significant impact on PAC frequency	No significant impact on PVC frequency
Obesity	Conflicting evidence	Higher PVC rates in cases of obesity
Gender	Conflicting evidence	Higher PVC rates in males
Hypertension	Correlation with PACS	Correlation with PVCs
Smoking	Increased frequency of PACS	Increased frequency of PVCs

Abbreviations: PAC, premature atrial contraction; PVC, premature ventricular contraction.

### 3.3 Caffeine

In the United States, 85% of the population consumes at least one caffeinated beverage daily. The average daily caffeine intake for all ages is 165 mg. Individuals aged 50–64 have the highest intake of 226 mg/day. The 90th percentile intake is 380 mg/day. Coffee is the main source of caffeine across all age groups, while younger individuals (<18 years) get a higher percentage of caffeine from carbonated soft drinks and tea.^36^ They also noted that in older ages, caffeine consumption was more common, and coffee was the main source of caffeine. Two studies by Mitchell et al.^36^ and Fulgoni et al.^37^ reported a similar prevalence (89% and 85%, respectively) of caffeine consumption in the US population. Additionally, Fulgoni et al.^37^ reported that the highest amount of caffeine consumption was in men aged 31–50 years, whereas the lowest amount was in women aged 19–30. The daily intake of caffeine per body weight was similar in men and women. A higher caffeine intake (≥400 mg/day) was more commonly observed in men. Caffeine consumption was more prevalent in single students, first‐year students, and those with higher grade point averages, and caffeinated food and beverages were more commonly consumed during exams and stressful times. Furthermore, the prevalence of high BMI was higher in the caffeine‐consuming group. According to the study by Lieberman et al.,^38^ age and ethnicity were the most associated variables with caffeine intake, smoking, working hours, and daily calorie intake being other associated factors.^37^, ^38^ They also reported that caffeine consumption was not significantly related to physical activity, economic status, education, or employment status. White non‐Hispanics and the middle‐aged group (50–54 years) had the highest caffeine consumption. Overall, caffeine consumption is more common among adolescents, smokers, and those of white ethnicity.

### 3.4 Caffeine and the risk of PAC and PVC

Marcus et al.^39^ conducted a trial that compared the prevalence of PAC and PVC between caffeine consumers and non‐consumers. They concluded that caffeine consumption and the prevalence of daily PAC were not related; however, higher caffeine intake was associated with a higher risk of PVC. Dixit et al.^1^ also concluded that there was no significant relationship between caffeine and PAC, but they also noted that PVC and caffeine intake are not related. In a multicenter study by Kim et al.,^40^ a higher amount of habitual caffeine intake led to a lower risk of cardiac arrhythmia.

## 4 CHALLENGES

Healthcare professionals facing extended shift hours and minimal room for error often experience significant demands, which can result in fatigue, disrupted sleep, and an increased likelihood of making mistakes.^41^ To address these challenges, many healthcare staff tend to consume caffeine as a solution for managing tiredness, coping with the stress associated with their jobs, and enhancing their alertness—all essential factors in their work environments.^42^, ^43^ Additionally, individuals working night shifts intentionally rely on caffeine to stay awake and remain sharp during their demanding duties.^44^

Like many other drugs and medications, caffeine has a range of potential side effects. These effects can span from relatively minor discomfort to potentially quite severe, and in some rare cases, even life‐threatening. The severity of these adverse effects is often linked to both the amount of caffeine consumed and individual sensitivity to the substance.^45^ Common side effects include anxiety, restlessness, fidgeting, insomnia, facial flushing, increased urination, muscle twitches or tremors, irritability, agitation, and elevated and even irregular heart rate.^11^

Furthermore, caffeine can stimulate the body's stress response system, resulting in increased release of stress hormones such as glucocorticoids and catecholamines, which in turn can lead to an increase in blood pressure. Therefore, caffeine consumption during stressful periods can extend and intensify the body's stress response, affecting both blood pressure and the release of stress hormones. This insight could have vital implications for individuals who are particularly sensitive to caffeine, especially those at risk of developing hypertension.^14^

Additionally, in individuals with heightened sensitivity to caffeine, consuming high doses can result in a set of symptoms collectively known as ‘caffeinism’. These symptoms may include increased anxiety, restlessness, nervousness, dysphoria, difficulties in falling asleep, and a tendency for thoughts and expressions to become disorganized and erratic.^46^

Among the limited number of studies highlighting a connection between coffee consumption and heart rhythm irregularities, one notable report revealed that a higher daily coffee intake was associated with a greater probability of PVCs observed during a 2‐min electrocardiogram recording.^30^ Moreover, an increased occurrence of PAC was found to be a predictor of the development of new‐onset atrial fibrillation. Remarkably, a greater prevalence of PVC was associated with an increased risk of heart failure.^39^ A noteworthy aspect in this context is that certain subgroups, particularly those with specific genetic variations such as CYP1A2 or COMT variants, may be more prone to the cardiovascular effects of caffeine.^47^

Several studies have consistently validated the safety of caffeine consumption at typical daily usage levels. Nevertheless, exceeding a specific caffeine threshold, approximately estimated at 1.2 g, can lead to undesirable side effects. These repercussions encompass symptoms such as an accelerated heart rate, irregular cardiac rhythms, altered mental function, and even seizures. In the most severe cases, excessively high caffeine doses, typically falling within the range of 10–14 g, have been associated with life‐threatening outcomes.^11^ Excessive caffeine intake appears to disrupt the natural electrical activity of the heart, resulting in the emergence of various arrhythmias. Therefore, individuals should consume moderate and measured coffee consumption to prevent the risk of these adverse consequences.^39^, ^48^

How individuals respond to caffeine can vary considerably, and this diversity is strongly shaped by the process of habituation and tolerance that develops in response to many of the physiological effects of caffeine. Those who are not accustomed to consuming caffeine regularly tend to avoid developing this habituation or tolerance. Consequently, they often feel the effects of caffeine even when their dietary intake is low.^49^

Moreover, the diversity in how caffeine affects individuals can be attributed to a range of genetic factors. Specific genetic variations, known as polymorphisms, have the potential to impact both the manner in which caffeine is metabolized in the body and its effects mediated by receptors.^45^ In addition, tolerance can develop as a response to the increased levels of tension, anxiety, and jitteriness that are often linked to the consumption of caffeine.^50^

The issue of caffeine dependence is a matter of significant concern. While caffeine is recognized as a mild stimulant of the central nervous system, preclinical studies have indicated that it does not trigger dopaminergic transmission in the nucleus accumbens shell, a distinctive characteristic observed in substances associated with addiction. Sudden cessation of caffeine intake can result in mild and transient withdrawal symptoms. These typically begin after 12–24 h of abstinence, peak around 20–48 h, and may persist for up to a week. These symptoms include headaches, fatigue, drowsiness, irritability, depressed mood, and anxiety. However, these withdrawal symptoms can be prevented by gradually reducing caffeine consumption rather than abruptly discontinuing it. In cases where symptoms do manifest, they can be quickly relieved by caffeine re‐administration. Importantly, caffeine withdrawal symptoms may vary significantly among individuals, but they are typically short‐lived and not harmful.^11^, ^51^

Caffeine acts as a “reinforcer,” easing withdrawal discomfort, but how it does so is not fully understood. Tea and coffee contain sufficient caffeine to serve as reinforcers; people often rely on these beverages during withdrawal, forming a cycle of dependency. Even a small amount, like 25–50 mg of caffeine per cup of coffee, can offer relief.^46^, ^52^

## 5 RECOMMENDATIONS

Caffeine's effects depend on the dosage, and high doses can impact cardiac contractility and increase the risk of arrhythmias.^53^, ^54^ Healthcare workers should prioritize following tailored guidelines that promote the safe consumption of caffeine and ensure that they do not exceed the recommended limits. The recommended daily caffeine limit for adults (aged 18 and older) is approximately 400 mg, which does not result in negative consequences.^40^, ^55^

Long work shifts cause sleep deprivation, affecting healthcare workers who often experience circadian misalignment and sleep inertia. They may surpass their limits,^56^ and as caffeine possesses qualities that enhance mental alertness, increase energy levels, and diminish feelings of sleepiness and fatigue, it can be a useful stimulant in situations requiring extended alertness and cognitive function, particularly when alertness is diminished.^56^, ^57^, ^58^ This can result in increased caffeine intake among healthcare workers with long shifts. Therefore, healthcare institutions should explore the adoption of flexible shift scheduling and work‐life balance initiatives. This move aims to decrease the dependence on caffeine among healthcare workers and, consequently, enhance the quality of patient care they provide.

Also, in a recent study, researchers examined the impact of substituting caffeinated or decaffeinated coffee with a non‐caffeinated coffee alternative on adverse caffeine effects. Findings suggest that individuals experiencing caffeine‐related problems, such as functional dyspepsia, may find relief by transitioning to a coffee substitute.^59^ Therefore, promoting the use of caffeine substitutes or alternatives and facilitating their accessibility within healthcare institutions may be a favorable approach.

Some studies have shown that in cases without structural heart disease, heightened stress and anxiety levels are associated with an increase in catecholamines, which in turn can lead to a more frequent occurrence of ectopic heartbeats in both ventricular and atrial arrhythmias.^60^, ^61^ On the other hand, caffeine may induce anxiety symptoms in the general population, especially at high doses. These effects tend to be more prominent in sensitive individuals, including those with panic disorder, depression, and social anxiety disorder.^62^, ^63^ Also, doctors often prioritize their altruistic and professional motives, potentially neglecting their well‐being. The impact on mental health, particularly anxiety disorders, is a notable concern in the context of hospital medicine.^64^ Given that anxiety can contribute to an increased occurrence of ectopic heartbeats, these individuals may face a heightened risk associated with the consumption of excessive caffeine. Therefore, the implementation of stress management programs and mental health support services for healthcare workers can be advantageous in this context.

Ultimately, there is a need for a multidisciplinary approach to reduce caffeine‐related PACs and PVCs in healthcare workers, which is crucial for their well‐being and the quality of patient care. Implementing these recommendations may substantially enhance the health and productivity of healthcare workers, yet further scientific investigation is warranted to support and refine these measures.

## AUTHOR CONTRIBUTIONS

**Maryam Abdolmaleki:** Conceptualization (lead); investigation (equal); project administration (lead); supervision (supporting); writing— original draft preparation (equal); writing—review & editing (equal). **Laya Ohadi:** Conceptualization (supporting); investigation (equal); methodology (lead); validation (lead); writing—original draft preparation (equal). **Faraz Changizi:** Investigation (equal); writing—original draft preparation (equal); writing—review & editing (equal). **Sara Seyed:** Investigation (equal); writing—original draft preparation (equal). **Mina Farjam:** Project administration (supporting); investigation (equal); supervision (lead); writing—original draft preparation (equal).

## CONFLICT OF INTEREST STATEMENT

The authors declare no conflicts of interest.

## TRANSPARENCY STATEMENT

The lead author Mina Farjam affirms that this manuscript is an honest, accurate, and transparent account of the study being reported; that no important aspects of the study have been omitted; and that any discrepancies from the study as planned (and, if relevant, registered) have been explained.

## Data Availability

Data sharing not applicable to this article as no datasets were generated or analyzed during the current study. The data and materials used in the current study are available from the corresponding author upon reasonable request.
